# Harmony and Distress: Humor, Culture, and Psychological Well-Being in South Korean Organizations

**DOI:** 10.3389/fpsyg.2018.02643

**Published:** 2019-01-07

**Authors:** Hee Sun Kim, Barbara A. Plester

**Affiliations:** ^1^Department of Business Administration, College of Government and Business, Yonsei University, Wonju, South Korea; ^2^Department of Management and International Business, The University of Auckland, Auckland, New Zealand

**Keywords:** confucianism, hierarchy, humor, psychological well-being, South Korea

## Abstract

Humor is a contextual phenomenon that exists in all societies, although the impact of humor may differ across different cultures. The data for this research was collected using an ethnographic-based approach, incorporating participant observation and semi-structured interviews. Based in three different South Korean organizations, this research offered the opportunity to interact in depth with workers of varying ages, genders, hierarchical levels, and organizational roles. Humor styles incorporate both light and dark forms of humor that may be affiliative and self-enhancing or aggressive and detrimental or may contain elements from both styles simultaneously Observations were complimented by 46 in-depth interviews and *ad hoc* follow-up discussions. This paper adopts a Confucian perspective to understand the perception and use of workplace humor in these South Korean organizations. Confucian philosophy emphasizes the value of formality within society in order to maintain respectful relationships between individuals. We suggest that humor is used in these South Korean workplaces to support traditional Confucian values of harmony and hierarchy, and the changing cultural values in these workplaces influence humor perceptions in complex ways. The Confucian value of harmony maintained through the idea of hierarchy creates pressure for organizational members, to accept and cope with contentious humor in the workplace. In particular, the clash between the need to maintain harmonious relationships in the workplace (through obedience toward superiors) and the desire to reject some types of humor highlights issues for younger organizational members. Internal conflict creates distress for younger employees who may hold more Westernized values and this affects their emotional, psychological well-being. This study presents a cross-cultural perspective to organizational humor, and suggests that humor may not always be a positive experience for organizational members, but instead may create psychological distress in some employees.

## Introduction

Humor is a common and important aspect of organizational interactions. Humor is often seen as a positive phenomenon within the organizational context, helping to improve the quality of interpersonal relationships between organizational members (Cooper, 2008), dissolve unproductive thinking patterns (Minsky, 1980), improve problem solving techniques, and increase creativity (Isen et al., 1987). Humor is a highly contextual and subjective phenomenon that may be interpreted differently by individuals and can create unintended and unexpected workplace outcomes that impact the psychological well-being of individuals. For the purposes of this paper we conceptualize psychological well-being as ‘qualities associated with positive psychological functioning’ (Huta and Waterman, 2014, p. 1430) and in this broad definition we include aspects of subjective experiences, emotions and cognitive appraisals. While humor interactions may help to diminish stress and relieve tension for workers (Morreall, 1986), humor may also increase distress and disharmony in workplace contexts (Plester, 2016).

Modern South Korean (hereinafter Korean) organizations and its members display strong traditional Confucian values (Deuchler, 1992), where individuals are encouraged to use honorifics and emphasize hierarchical relationship structures in the workplace (McBrian, 1978). This influence of Confucian philosophy may affect the style of humor that is adopted, how humor is used, and how it is interpreted within Korean workplaces. This paper examines how the Confucian based values of hierarchy, formality, and respect, govern the use of humor in Korean organizations, and subsequently influence the psychological well-being of workers. We pay particular attention to younger workers, lower in the organizational hierarchy as our data indicates that Western influences are changing their perceptions of traditional organizational social practices. We question how workplace humor influences psychological well-being for employees in non-Western organizational contexts. This paper provides an original perspective into organizational humor within a Confucian context and presents implications for the psychological well-being of organizational members.

### Humor, Culture and Psychological Well-Being

In this review we examine the literature in three sections. Firstly, we present workplace humor literature and highlight its implications in different cultural contexts. We note that there are very few organizational studies that investigate humor from cultural perspectives other than studies derived from Western contexts. Then we shift our focus specifically to the Confucian-based work context and we discuss the hierarchical nature of Confucian traditions within Korean businesses. We conclude our review by evaluating literature pertaining to humor and psychological well-being.

### Humor at Work

Humor is a universal phenomenon that exists across all cultures (Berger, 1987). Humor is often considered a form of communication that creates a sense of commonality among people, due to the similar process of humor appreciation in human beings (Alden et al., 1993), and commonly relates to a perception of ‘fun,’ smile, and laughter (Ruch, 2008). Humor is a complex and contextual phenomenon, and is difficult to define (Alden et al., 1993). From a psychological perspective, the use of specific humor styles may help to describe individual differences in terms of personality, character, and ability (Ruch et al., 2018a), but also facilitate various interpersonal communicative intent in the workplace (Holmes, 2000). However, the same humor interaction may be interpreted differently by individuals (Ruch and Hehl, 2007) which can lead to unpredictable outcomes. Yet, humor is most commonly seen as a positive phenomenon that creates a feeling of happiness (La Fave et al., 1976) and is frequently encouraged in workplaces without consideration for potential (negative) impacts (Morreall, 1983). Plester (2016) definition, based on workplace research emphasizes the complex, contextual, and multi-faceted nature of humor and present humor as a complex social interaction that may incorporate feelings of amusement or can evoke laughter, but may also have unexpected or unpleasant (i.e., culture and implicit humor attitudes, Jiang et al., 2011) outcomes. However, to offer psychological focus we adopt Martin and Ford’s recent definition whereby they draw upon earlier work by Ruch (1998):

Humor is a broad, multifaceted term that represents anything that people say or do that others perceive as funny and tends to make them laugh, as well as the mental processes that go into both creating and perceiving such an amusing stimulus, and also the emotional response of mirth involved in the enjoyment of it (Martin and Ford, 2018, p. 3).

Organizational humor has been gaining more attention within organizational studies, but it is notable that these studies are mainly focused on Western organizational contexts. Collinson (1988) and Plester (2007, 2009, 2016) examine humor within British and New Zealand companies. Based in Canada, Rod Martin’s many studies into humor and psychological well-being, although multi-dimensional, are conducted with Western theoretical conceptions and underpinnings. The late sociologist Christie Davies explains the motivation for ethnic jokes, and does include a chapter on Anglo Saxon attitudes to humor but does not investigate humor from non-Western cultural contexts. Cooper (2008) and Romero and Pescosolido (2008) both offer comprehensive conceptual analyses of humor and its impact on work relations, group processes and leadership but all from Western perspectives based on analyses of Western studies.

Furthermore, there are limited studies that focus on humor and culture in ethnography field (i.e., Powers, 1994), and many cross-cultural humor studies tend to examine humor in a quantitative manner (e.g., Kalliny et al., 2006), which limits our understanding of humor in diverse contexts. Although humor is normative and highly influenced by each organizational culture (Plester, 2016), humor may also be affected by the society in which the organization is based. For example, Davis (2016) study of humor and satire in Australia, Japan, and China illustrates the complexity of humor (satire) recognition and reactions in different cultures and timeframes. Because humor is relational and contextual, language and norms adopted by individuals involved in humor exchanges can impact how humor is used and interpreted.

From a functional perspective, humor may help to define the cultural identity of individuals, reinforce social norms (Bricker, 1980; Duncan, 1985), and power relations within a group (Fine and Soucey, 2005; Huber and Brown, 2016). Through the use of humor, individuals may identify similarities with others via laughter, and thus establish familiarity between diverse groups of people (Brown and Levinson, 1978). However, humor may also disrupt the flow of organizational communication and damage workplace relationships by creating ambiguity and misunderstanding between the communicators, while some inappropriate or contentious humor may result in negative relational outcomes. Humor styles may vary from ‘light’ humor that may be fun, affiliative and self-enhancing to ‘dark’ forms of humor that can be aggressive and detrimental (see Martin, 2003) and some humor instances may even incorporate both styles of humor at the same time.

Cooper’s (2008) model depicts social processes that function in conjunction with the individual-level mechanisms in humor. Cooper suggests that interpersonal humor operates through four related processes of affect-reinforcement, similarity-attraction, self-disclosure, and hierarchical salience. Affect-reinforcement suggests that a positive experience helps to establish a feeling of attraction between interacting individuals. Since using humor can arouse emotions in people, a greater level of affection may be achieved if individuals enjoy the humor and the interactions with the joker. This is because humor is a form of social communication which reinforces or punishes the behavior (such as enjoyable behavior) of interacting individuals (Baron, 1984). Perceived similarity is the degree to which an individual believes that s/he is similar to a target individual. Individuals that find the same humor event enjoyable may be attracted to each other, as this enjoyment implies that the individuals share similar attitudes and beliefs (Byrne, 1971). Sharing humor in the workplace is a form of self-disclosure, as it exposes part of individual’s (joker’s) information (for example, their humor preferences).

Voluntarily engaging in light humor may lead to people feeling closer to one another (Collins and Miller, 1994), as humor is not usually required for work. However, dark humor may have a negative effect if it violates the beliefs and norms of communicating individuals (Derlega et al., 1993). For example, hierarchical factors can influence workplace humor, and this can be complicated as the different organizational positions may be perceived differently to each communicating individual. While using humor can enhance the relationship between individuals, it can have the opposite effect when shared between those at a different level of hierarchy. The authoritative distinction between organizational members may be highlighted if a manager uses humor to control the behavior of employees (Martineau, 1972; Holmes, 2000). In contrast, interpersonal barriers crafted by the formal organizational hierarchy may be dissolved through light successful humor (Vinton, 1989). Subordinates can use the ‘safety shield’ and ambiguous nature of humor to challenge managers and express disagreements (Holmes, 2000; Plester and Orams, 2008).

However, the idea of humor may be approached differently in certain cultures (Alden et al., 1993). For example, Yue et al. (2016) suggested that within their study, Chinese people consider humor as less valuable than Canadians, and also considered themselves (Chinese) as less humorous people. This may be because China is one of the East Asian cultures that have historically been influenced by Confucian philosophy, which prescribe strong belief toward respect, hierarchy, and formality (Yao, 2000), and influencing how individuals view others, and themselves. Under Confucian assumptions, humor may be perceived as an inappropriate form of communication, especially as it may damage the authority of the manager with higher organizational status. Davis and Chey’s (2013) study on Chinese life and humor implies that culture-specific codes such as structure, medium, topics, and styles should be carefully considered as a part of the environmental factors when examining humor and culture. This means that the use of humor in different cultural contexts may be risky, as engaging in humor without considering such cultural assumptions may have some unintentional and unpredictable effects.

### Confucianism at Work

Confucianism is a philosophy of ethics that values interdependency and harmony amongst individuals (Deuchler, 1992; Yao, 2000). Confucian values, which originate from the Chinese scholar Confucius have historically affected many East-Asian cultures such as China, Japan, Vietnam, and Korea (Duncan, 2002). Confucianism emphasizes the role of hierarchy and formality, which is maintained through structured communication and everyday rituals and rites (Yao, 2000).

Under Confucian assumptions of hierarchy and formality, humor may be an unsuitable form of communication to use within the workplace. Cultures that embed Confucian values perceive humor differently to those in Western contexts. Yue et al. (2016) suggests that the idea of humor is approached differently between Western and Eastern contexts, and that Confucian cultures may not favor humor in social interactions, whereas humor interactions are more common within Western contexts. In particular, the unequal relationship structure prescribed under Confucianism influences the communication process between individuals of different hierarchical levels, and thus silence tends to be the preferred mode of communication for those in subordinate positions (Lim, 1999). However, even with such strong behavioral norms, humor still occurs in Confucian-based contexts such as Korea (Kim and Lee, 2009; Jung, 2014). For example, Jung (2014) study on Korean public organizations displayed frequent use of humor within the workplace, especially by those in senior positions. This suggests that while Confucian values may influence humor interactions of individuals within Korean organizations, this influence may be displayed in complex ways and seems significantly linked to hierarchical dynamics. Therefore, it seems that senior employees may have greater license to display and use humor in Korean organizations.

Korean society maintains strong Confucian based cultural traditions, reinforced through language, norms, and rituals (Deuchler, 1992). While Confucianism is not the only philosophy that historically influenced the Korean people, modern Korean society displays strong Confucian traditions that shapes the relationship structure and communication between individuals (Choi, 2010), similar to other East Asian cultures such as Japan, Vietnam, and China (Duncan, 2002). Despite the cultural difference between different generations within the Korean society (Ingelhart, 1997), the general Korean population, including the younger generations still strongly endorse the Confucian value of *harmony* (Zhang et al., 2005).

Confucian values in Korea are reinforced consistently through everyday practices and language, to emphasize respect and hierarchy (Deuchler, 1992). These hierarchical principles spill into the organizational context (Rowley and Bae, 2003), and natural inequality and hierarchy determine the organizational communication and relationships. Individuals are expected to reflect the hierarchical relationship structures by behaving according to their relative status (Clark, 2000), and use appropriate verbal (honorifics, McBrian, 1978) and physical (bowing, Cho and Yoon, 2001) language. Based on this hierarchical relationship structure, silence is considered the preferred form of communication for individuals with lower hierarchical status (Lim, 1999), to show respect (Yuan, 2015), obedience (Song and Meek, 1998), and contribute toward group harmony (Barnlund and Yoshioka, 1990). Therefore, under such Confucian based assumptions, humor may be considered inappropriate in the workplace.

Cooper’s relational process model (Cooper, 2008) suggests that humor may influence the quality of interpersonal relationships between organizational members. This includes the relationship between individuals at different levels of organizational hierarchy, and she suggests that humor may help to diminish the psychological distance within superior-subordinate relationships. However, this humor dynamic may be problematic in Confucian contexts, where hierarchy is believed to be an important part of maintaining harmony and order within a community (Yao, 2000). Under this hierarchical perspective, humor may be considered too ambiguous and sometimes even a dangerous form of communication that may damage organizational relationships (especially in superior-subordinate relationships) by being perceived as a challenge to the hierarchical relationship structure. This potential risk of humor may impact the psychological well-being of individuals at lower hierarchical levels.

### Psychological Well-Being and Humor

Psychological well-being is important and so organizations have become more conscious about this important aspect of employees’ workplace experiences. Psychological well-being is a broad concept that concerns the mind-set of individuals and how people evaluate and judge the quality of their lives (Keyes, 2007). Psychological well-being refers to a person’s view about their functionalities, capacities, performance, and social relations, and includes the subjective experiences, emotions and cognitive appraisals of individuals (Huta and Waterman, 2014).

Psychological well-being is a popular topic in current management literature, especially as people are becoming more aware of the importance of emotional and psychological health issues in the workplace. This is because psychological well-being is related to workplace stress (Folkman and Lazarus, 1988; Papousek, 2018) and absence of distress (Huta and Waterman, 2014) which may affect individuals’ self-esteem (Berger et al., 2011), job satisfaction (Wright, 2004, 2005), and burnout (Schaufeli et al., 1993). Furthermore, low emotional and psychological well-being of individuals may lead to a high level of turnover (Wright and Bonett, 2007).

This literature suggests that it is important for individuals to maintain a positive psychological state within their workgroup as a healthy psychological state will help to maintain a positive work life, and from an organizational perspective, which can help employees to achieve high performance. Therefore, it is important for organizations to recognize ways of relieving work stress and help employees recover and maintain their well-being (Demerouti et al., 2011; Sonnentag et al., 2013).

Both light and dark forms of humor may help individuals to relieve stress and tension. A Freudian notion of humor suggests that people use humor to relieve emotions and desires that may be considered antisocial (Freud, 1960). By using humor as a mechanism to relieve strong feelings (such as anger) people may safely release internal tensions while still observing the social norms and behavior. Therefore, humor helps to reduce stress and helps people to manage their psychological well-being (Martin et al., 2009). High levels of humor are associated with a more positive and self-protective stance when individuals experience stress, and humor helps people to respond more positively to both positive and negative life events, enhancing their psychological well-being (Martin et al., 2009; Papousek, 2018).

Light forms of humor such as Crawford and Caltabiano’s (2011) experimental study suggests that humor helps to improve individuals’ self-efficacy, positive affect, optimism, and perceptions of control, while decreasing anxiety, depression, and perceived stress, to enhance the overall psychological well-being of individuals. Erickson and Feldstein (2007) also suggest that humor may help individuals to cope and relieve psychological distress, to maintain a clinically healthy state of well-being. However, humor may not always create *positive* affects for health and emotional well-being.

Humor is a social process which affects those involved in the humor interaction, including the joke creator (joker), the target of the humor, and audience or observers to the humor (Plester and Inkson, 2018). This means while humor may allow, for example, the joker to relieve stress, others involved in the joke may react differently and experience the humor as ‘unfunny’ or even offensive (Plester, 2016). We frame this type of humor as ‘dark’ in line with Martin (2003) humor styles inventory and note that such humor can cause distress. In a hierarchical relationship within workplaces, where it is difficult for subordinate employees to express any discomfort about dark humor, employee stress may be increased. Plester (2016) suggests that the ‘dark side’ to humor does not always create happiness and positive effects in the workplace but can damage the relationship between work colleagues and also cause emotional or psychological harm. Negative social outcomes, such as offensive humor interactions, may have an even stronger impact than positive experiences, on psychological well-being (Rook, 1984).

While we acknowledge that the use of ‘light’ affiliative humor may lead to higher levels of social self-esteem, lower levels of depression, and thus contribute to an individual’s level of psychological well-being (Kuiper and McHale, 2009) it is important to keep the potential negative impacts in sight. Martin et al. (2003) developed a humor styles questionnaire which helps to explore the relationship between humor and psychological well-being by analyzing types of humor that people use. The humor styles questionnaire balances positive aspects with assessment of how people use humor in less-desirable ways that have the potential to damage the psychological well-being of others, especially through ‘dark,’ aggressive and self-defeating humor. Similarly, Ruch et al. (2018b) suggests that light humor styles relate to greater psychological well-being of individuals, while dark styles including mockery, cynicism and sarcasm relate to lower psychological well-being. Research from both Ruch et al. (2018b) and Martin (2003) highlights the influence of specific humor styles on the well-being of individuals.

Within organizational relationships, supervisors’ behavior strongly influences the psychological well-being of employees, much more than the behavioral influence of other organizational members (Gilbreath and Benson, 2004). Kuiper and Martin (1998) emphasize humor as an intrapersonal adaptive coping strategy and so it can be used in challenging or threatening situations which reduces the risk of being overwhelmed by negative emotion (Lefcourt and Davidson-Katz, 1991). Such humor use can somewhat protect lower ranking employees who may use humor to cope with challenging or threatening situations involving their supervisors, and so they defend their psychological well-being. However, this effect does not necessarily apply to those who become the subject (victim) of dark humor and becoming the target of a joke, jibe or prank can be psychologically and emotionally destructive (for examples see Plester, 2015, 2016). Within cultural contexts (for our purposes -Korea) traditionally influenced by Confucian values of hierarchy and harmony, humor may not achieve positive psychological outcomes especially for those in subordinate positions, as humor use is not a form of communication or a coping strategy that aligns with Confucian ideals (Lim, 1999).

In order to understand the influence of humor on the psychological well-being of individuals within Confucian-based workplaces such as Korea, measuring humor styles as suggested by Martin et al. (2003) does not provide the rich contextual understanding of the humor interaction, and will not explain the different interpretations of workplace humor for those influenced by Confucianism (Yue et al., 2016). Therefore, this current study attempts to provide a cultural understanding of organizational humor, examining the relationship between humor and psychological well-being in Korean organizations. Our research question asks: how does workplace humor influence psychological well-being for employees in non-Western organizational contexts? To answer this question, we adopt an ethnographic-based approach method, discussed next.

## Methodology

### Multi-Voiced Interpretivist Approach

This exploratory research adopts a qualitative approach to investigate the complex relationship between organizational humor, Confucian cultural values, and psychological well-being of employees. We examine these dynamics in depth by collecting the subjective perceptions and stories of individuals through observations, interviews and conversations. As humor is a social process that may create different meanings for each interacting individual (Holmes, 2006), and meanings can change depending on the particular context of interaction, humor needs to be examined through multiple interpretations. Therefore, a multi-voiced interpretivist approach (Alvesson, 2010; Cunliffe et al., 2014) is used to explore the diverse interpretations and impact of humor within our organizations, and we reflect upon humor as a contextual phenomenon (Kalliny et al., 2006). This approach helps to illustrate multiple stories with regards to humor, culture, and psychological well-being within Korean organizations. However, this qualitative study does not attempt to generalize the findings, and acknowledges the idiosyncratic nature of the resulting data. Instead, this research intends to illustrate unique situations and rich descriptions of the studied companies, in attempt to understand the complex relationships and processes questioned (Bryman and Bell, 2015).

### Data Collection Methods

We used an ethnographic approach (therefore *not* ethnography) to investigate three Korean organizations across the period of March 2014 to June 2014. We initially intended to undertake participant observation, semi-structured interviews, and document collection to capture individual interpretations of humor interactions. It was also our intention to collect any documentation related to humor and employee psychological well-being but unfortunately only one of the three participant companies had documentation regarding employee well-being, and none with regards to humor. The collected employee well-being documentation discussed methods and recommendations for organizational members to maintain a happy work life (‘to do lists’) for themselves and also for others. This document was a small manual-like booklet (A5 size booklet, 12 pages, including illustrations, figures, and tables) issued by a ‘meditation group’ that the company’s CEO actively participate in. Therefore, this avenue of data collection was only viable in one of our studied companies and does not have a strong influence on our results and discussion.

The three participant companies each have unique characteristics, culture, and are of different sizes. These companies were selected based on the assumption that these diverse contexts will help to provide greatest learning about the studied phenomena (Stake, 2013). Each organization operates in a different industry including manufacturing, online gaming, and Information Technology (IT). For the purpose of this research, the participant companies have been re-named Wisepath, Mintrack, and Truscene. All individual participants have also been re-named using pseudonyms. Table 1 below summarizes the participant companies and denotes the organizational hierarchy levels examined in this study.

**Table 1:** Summary of the participant companies.

Company	Industry	Size (no. of workers)	Levels of organizational hierarchy	Age range
Wisepath	Manufacturing	63	10	Early 20’s to mid 60’s
Mintrack	Online Gaming	33	8	Early 20’s to mid 60’s
Truscene	IT	49	7	Early 20’s to mid 40’s

In order to capture natural day-to-day humor interactions in the workplace, one researcher spent a calendar month of full immersion in each company. She participated in most organizational activities (both formal and informal) that she was able to, and these included activities within and outside working hours, and sometimes weekends. In efforts to ‘fit in’ our researcher also engaged in some work tasks, including translating organizational documents, interpreting (English) in company events, and dismantling devices in factories. Furthermore, as she is ethnically Korean and speaks Korean fluently, this helped the participants to engage with the researcher more easily and carry out in-depth discussions (Temple and Young, 2004). All research participants were provided with information with regards to the research, and participation was voluntary. All members within the studied companies agreed to participate in the research (observation), and 46 individuals agreed to be interviewed. However, the demographics of these 46 interviewees do not proportionately represent each of the participating companies. Therefore, 46 semi-structured interviews were conducted across the three participating companies. Questions with regards to the use of humor, perceptions toward humor, cultural values and expectations within and outside the workplace, and individual’s psychological well-being in relation to such perceptions and behaviors were discussed in the interview. Throughout the study we observed humor interactions that we interpreted as significant. We acknowledge that this was based on researcher interpretation based on factor such as obvious emotional reactions by participants (such as loud obvious laughter or alternatively signs of distress such as a po-faced response or expressions that seemed to indicate any displeasure). When our interpretations indicated a significant incident we further explored peoples’ reactions by conducting *ad hoc* interviews that allowed them to discuss their feelings about the observed interaction. Demographic details on these 46 interview participants are summarized in Table 2.

**Table 2:** Summary of interview participants.

Company	No. of interview participants	Gender	Age range	Hierarchical status
Wisepath	7	5 male, 2 female	Early 30’s to mid 60’s	4 staff (level 1), 2 deputy managing director (level 7), 1 managing director (level 8)
Mintrack	14	10 male, 4 female	Mid 20’s to early 40’s	6 staff (level 1), 1 manager (level 2), 2 deputy section chief (level 3), 3 section chief (level 4), 1 deputy department manager (level 5), 1 department manager (level 6)
Truscene	25	20 male, 5 female	Early 20’s to mid 40’s	10 staff (level 1), 5 manager (level 2), 3 deputy section chief (level 3), 3 section chief (level 4), 2 deputy department manager (level 5), 1 department manager (level 6), 1 senior management (level 7)

**FIGURE 1:**
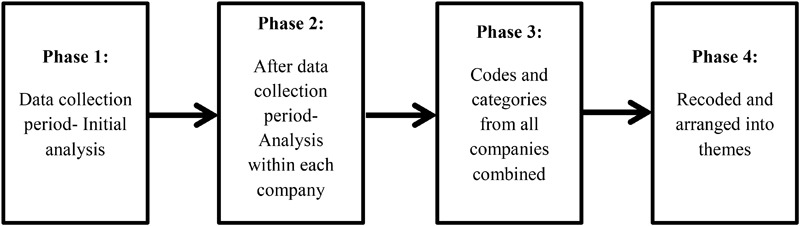
Phases of analysis.

Thematic analysis is used to code and organize the data. Thematic analysis provides flexibility to analyze the collected data, which fits with the explorative nature of this research (Braun and Clarke, 2006). The analysis focuses upon the humor interactions between individuals of different hierarchical levels, differences in individual interpretations, and any perceived emotional impacts on employees. These were analyzed by carefully examining similar key words or meanings explained in individual interviews, and in observation data that reflected such ideas (Auerbach and Silverstein, 2003). Similarities and differences in humor interactions within and across the three participant companies were investigated, by grouping observed interactions and also interview excerpts that support (or sometimes provide alternative interpretations to) a particular idea. This process of data analysis was repeated and conducted in multiple stages to craft different themes and categories. Figure 1 illustrates the different analysis phases used in this research.

We conducted initial analysis during the data collection period, followed by analyzing each participant company’s data as a whole after the 1 month of observation period. Then the data from all three participant companies were organized and combined using NVivo. Therefore, data was coded in multiple stages and points in time. Coding was conducted and repeated throughout data collection period, transcription period, and (post-data organization) ‘data analysis’ period. A reflective diary was recorded throughout the data collection period, in order to incorporate reflexivity throughout the research process. This included notes on observations and interviews, and personal reflections on each of instances, which helped the researcher to maintain an open mind about the interpretation of data. This process helped to provide an in-depth understanding of organizational humor in the Korean context, and three themes and eight categories emerged in this process. These themes and categories are explained in the findings section.

### The Context

In order to understand the data presented in this research, it is important to recognize the context in which the data was collected. This section explains the Confucian-based relationships observed within each of the participant companies, illustrated through language and observed behaviors of the participants. Employees from all three participant companies displayed organizational humor in a variety of everyday interactions. While each of these participant companies had a different organizational culture, the Confucian-based values of respect and hierarchy were consistent across the companies. These were displayed through language (honorifics and titles- honorifics are commonly displayed in East Asian countries such as Korean and Japan, in communication by using specific grammar and phrases that shows respect and hierarchical differences between the communicators), communication (gestures), and organizational hierarchy and discussed in interviews.

#### Wisepath: Manufacturing Company

Manager and employees at Wisepath used the most formal and hierarchical communication, where strict honorifics were used by the subordinates to their managers. However, managers did not use any honorifics or organizational positions to address subordinates. Only the subordinate’s first name was used by the managers which denote significant hierarchical difference between managers and employees. This was a norm in this particular organization’s interactions.

#### Truscene: Information Technology Company

At Truscene hierarchy was much embedded in formality and protocol. Honorifics were used by most organizational members, regardless of their organizational position. However, subordinates used the term ‘nim’ toward their superiors to show respect. The term ‘nim’ translates to ‘sir’ or ‘madam’ in English, and ‘sama’ in Japanese and it emphasizes the heightened status of the manager. This was the only participant company that had documentation about employee’s psychological well-being. However, while they seemed to care about employees’ psychological well-being, observations show that they also had strict, formally documented rules in place that were designed to maintain a calm, collective workgroup where individual needs were secondary. Documents included a list of procedures stipulating that employees must remain calm in conflicting situations (with customers or work colleagues) and some guidelines on ways to communicate in order to achieve a happy work life (for self and others). A training program was also provided for all employees, to teach skills for maintaining a calm state of mind (this is not meditation). This involved role-playing to understand the feeling of others (colleagues and customers), and ‘emotional training’ to help increase sympathy toward others. Observations showed that employees of lower hierarchical status were trained to behave in this manner in order to build harmony and happiness in the collective group, and to accept managerial decisions calmly and unquestioningly. Held on a Saturday morning, this observation record demonstrates the ‘emotional training’:

19 senior employees sit around in a circle. It is just past 4:00 pm, and everyone looks tired, but keeps a smile on their face. The CEO asks the group to make any comments or give him an ‘honest piece of mind’ so that he can understand the thoughts and feelings of the workers more. Everyone remains silent. After a few minutes, Rubelle starts to talk.

Rubelle: I think it is sometimes inappropriate, where the CEO, sir, mentions about another person’s looks or their behavior. Sometimes that could hurt them, and I thought that could be an issue we can discuss today. Just thinking about how others could become emotionally hurt by another person’s light comment.

The CEO remains quiet, frowns, rubs around his mouth, and asks the group to provide other feedback. Four different managers start to talk at the same time, criticizing Rubelle for his thoughtless comment.

Lori: Did you consider how the CEO would feel, after you say something like that? The CEO probably made that comment to help us improve our abilities, and to become a better person. I’m surprised that you were thinking that way.

CEO: I’m quite surprised too. And that you were thinking like that, about me.

Several other managers make a comment to Rubelle simultaneously, making a loud noise. Rubelle sits with his hands folded together, looking down, not making any further comments.

March 28, Truscene Observation notes

This observation suggests that although Truscene managers express concern about the psychological and emotional wellbeing of workers, the training involved and cultural norms developed through these processes reinforces hierarchy only. Other managers that were observing Rubelle’s action (seen as a criticism of the CEO’s behavior) quickly correct Rubelle, to reinforce the social norm of respecting superiors (managers), even if it harms Rubelle’s feelings and emotions. Similarly, booklets and information about psychological well-being of the employees were more concerned with understanding the emotional needs of others, and with repressing excessive emotion, which is perceived negatively. Although this observation is lacking in humor, it does significantly show the hierarchical, patriarchal context of the studied Confucian organizations.

#### Mintrack: Online Gaming Company

Mintrack was less formal in terms of the language used by the organizational members. Although individuals in subordinate positions used honorifics when communicating with their superiors, family like titles were used to suggest a less formal and hierarchical relationship between the organizational members. For example, the term ‘unni’ (direct translation, ‘older sister’) was used instead of a work title. This suggests that although Mintrack is less formal in terms of communication it still maintains hierarchy by using personal-based titles that reinforce age-based hierarchical divisions albeit in a more familial way.

## Findings

### Themes and Categories

Data collected across the three participating companies were coded and analyzed to result in three overarching themes. Initial coding schemes for observations were developed based on humor and Confucianism literature. 17 coders were initially used to guide the observation process (refer to Sharpe and Koperwas, 2003) which focused on the hierarchical relationships, direction of humor use, types of humor, and emotional responses of individuals. While these codes were initially used to help categorize the collected data, further codes were developed in the midst of data collection period in order to remain open to new observations (Braun and Clarke, 2006). Forty two codes were developed in total, and these codes were then analyzed further to be grouped into eight categories and three themes. While some of these codes reflected conflicting behaviors from participants, these codes helped to provide an alternative interpretation to the behaviors of participants, which were also supported by follow up interviews. Table 3 below summarizes the different themes and categories derived through the data.

**Table 3:** Themes and categories of thematic analysis.

Themes	Categories	Category description	Examples
1. Hierarchical limits	(1a) Fulfill relative roles (41 sources, 142 references)	Use or response to humor interaction is perceived to fulfill the relative role (superior/subordinate) of the interacting individuals.	‘There’s this line (about roles). If you go over this line, it can be dangerous’ (Truscene)
	(1b) Demographic division (24 sources, 84 references)	Humor interaction is perceived to create a division between demographic groups (age, organizational hierarchy, gender), by using humor differently or contents of humor used creating such a divide.	‘Once I got told off by a passing-by manager for joking around with someone […] he asked who has the longer tenure, and only when I told him that we were only a month apart (in organizational tenure) he backed off’ (Truscene)
	(1c) Confucian values (14 sources, 26 references)	Workplace humor is perceived to be somewhat influenced by Confucian values	‘How we talk is based on hierarchy. Hierarchy is based on Confucian values’ (Wisepath)
2. Being respectful and preserving harmony	(2a) Maintain sound relationships (40 sources, 105 references)	The use of humor is perceived to maintain harmonious workplace relationships, regardless of its actual impact on individuals	‘Teasing and name-calling is similar to asking how your day has been. So I think saying things like this is a sign of care and closeness (by superior)’ (Mintrack)
	(2b) Being respectful (20 sources, 45 references)	The use and response to humor is perceived to reflect a (Confucian-based) respectful behavior	‘It comes from the traditional Yang-ban (aristocrat) behavior, being respectful to your seniors […] even humor should show respect, being rude cannot be accepted in Korean culture’ (Wisepath)
	(2c) ‘Good-is-good’ (21 sources, 32 references)	Any seemingly positive communication (i.e., humor) is perceived to help preserve workplace relationships	‘You can’t spit on a smiling face’ (Mintrack)
3. Negative emotions	(3a) Feeling hurt and rejected (21 sources, 60 references)	Experience of humor creates hurt feelings or some form of rejection for the individual	‘People keep on saying that I’m a stupid, but a nice person […] it doesn’t really feel good, but I do laugh it off... it doesn’t feel good’ (Mintrack)
	(3b) Stress and discomfort (20 sources, 33 references)	Direct or indirect (observed) humor experience creates some form of discomfort for the individual	‘It makes me feel anxious. Sometimes people overdo jokes, and it’s mischievous, and confusing’ (Mintrack)

The first theme *hierarchical limits* (categories: *fulfill relative roles, demographic division, Confucian values*) discuss how the use and perception toward workplace humor may be influenced by the hierarchical position of the interacting individuals. The emerging codes related to the relative role of superiors and subordinates (in various demographic and organizational groups), and how these role expectations may be implied through humor interactions to express some form of a respectful divide between groups. The second theme *being respectful and preserving harmony* (categories: *maintain sound relationships, being respectful, ‘good-is-good’*) discuss how workplace humor may be perceived to maintain harmonious relationships in the workplace, by behaving and responding respectfully to others’ use of humor. These categories focus on the idea of smiling and accepting (sometimes difficult) humor, in order to show respect and peace. The last theme *negative emotions* (categories: *feeling hurt and rejected, stress and discomfort*) discuss how the experience of humor may lead to (intentionally or unintentionally) unhappy feelings or negative emotions. Sub-codes within this theme included diverse descriptions of emotions experienced by participants (e.g., depression). Two different groups of codes emerged, where one group imply more temporal negativity and discomfort, and another implying emotional impact on the individual which somewhat extends outside work.

### Hierarchical Limits on Humor

Due to the use of honorifics and different titles toward individuals of superior status, communication for those in subordinate positions is highly complex. Observations show that the use of humor by employees in subordinate positions was limited and especially within subordinate-superior relationships. Across the three participant companies, 163 humor instances by a superior (to a subordinate) were observed. These humor instances displayed a mixture of different humor contents, target(s) of humor, and reactions of interacting parties. In contrast, only 40 humor instances by subordinates (to a superior) were observed. Table 4 below summarizes the observed humor directions, contents, target, and emotional reactions of individuals involved in the observed humor instances.

**Table 4:** Summary of observed humor instances.

Humor direction (observed instances)			
**Superior to subordinate**		**Subordinate to superior**	
163		40	
**Contents**			
**Interpersonal**	**Work**	**Interpersonal**	**Work**
124	39	35	5
**Target**			
Self/ subordinate/ third party	Self/ subordinate/ third party	Self/ third-party	Self
**Emotional reaction**			
Positive	Positive	Positive/ none/ negative	Positive/ none/ negative

Contents of humor presented in Table 4 above is categorized into the broad areas of interpersonal and work-related issues. Interpersonal category includes general non-work related jokes, word-play, and slapsticks (which may mimic humor of famous comedians, or those commonly known through the media). Furthermore, emotional reactions category in Table 4 displays the initial reactions of individuals involved in the observed humor instances, which are positive (any positive reactions such as a smile, including visible laughter), none (no reaction or participants leaving the scene immediately after the humor instance), and negative. We note that these may not be the actual emotions experienced by the individuals involved.

The observed humor instances suggest that humor initiated by superiors are more dynamic in terms of humor contents and targets subject to humor. A variety of contents, including work tasks, interpersonal issues, gendered and sexual humor were used by superiors, and targets range from self (joker), any other organizational member with lower hierarchical status (than the joker), to third parties. However, most humor instances by subordinates tended to focus on interpersonal issues or simply mimic word-plays trending in media, and thus being self-targeted in their humor. Emotional reactions displayed by other interacting individuals were distinctively different, where all subordinates reacted positively to a superior’s humor. Superiors responded either positively, negatively, or did not show any reaction (silence or leaving the scene) to subordinate’s humor. This observation on humor direction, contents, target, and emotional reaction was similar across the three participant companies. However, due to the nature of this research, the collected data do not proportionately represent these three companies (i.e., equal number of observed humor instances per company).

Although the number of observed humor instances by those in subordinate positions was limited, many of the younger participants in subordinate roles saw humor as a favorable phenomenon and stated that they enjoy using humor in the workplace. Five of these participants thought that humor creates a favorable atmosphere, and 13 participants stated that humor helps to relieve stress in the workplace. All of these participants were under the age of 30, and occupied positions low in their organizational hierarchy:

I always use humor to ease tension and to make people laugh.

25–29, Mintrack

I guess I could think favorably of that person, because it’s funny.

20–24, Truscene

While these younger participants claimed that humor at work had positive outcomes such as stress relief and good feelings, they also had experienced times where their managers’ humor had made them feel awkward and uncomfortable. They gave examples of times that they felt obligated to respond to (not so funny) humor -regardless of their true feelings:

You laugh because that’s what you need to do. A protocol. If it’s your senior, why do you even need to think about it? Just laugh, and everything will be fine.

30–34, Mintrack

This shows that humor is experienced differently according to the relative hierarchical differences between the work colleagues. The experience of stress creation or relief through humor may differ according to the hierarchical dynamic determined by organizational position, age, or a combination of both. This may create a form of demographic division (category 1b ‘demographic division,’ Table 3) within the workplace. This conversation between two young female workers articulates how humor may create further distress for workers, depending on hierarchy:

During lunch time, the CEO’s secretary and administration worker chat while eating their lunch in the company cafeteria.

Secretary: You remember when I went for my break, for my overseas trip that I’ve been planning for ages?

Administrator: Yeah I remember

Secretary: When I came back, the CEO was like, ‘where’s my gift? It was my birthday while you were gone. I thought you would’ve at least gotten me a gift, if you were leaving me to work by myself on my birthday’

Administrator: Oh my gosh, so what did you say?

Secretary: I apologized and laughed, but it was so awkward. He was joking, but you know, people like us can’t take that just as a joke. It was so uncomfortable. It stressed me out for the whole week, thinking that whether I should’ve gotten him a gift. But it was my break, and I am legally allowed to take time off, and why should I care about when his birthday was? I think I lost some hair (from stress).

May 27, Wisepath observation notes

This observation suggests that regardless of the intention of the CEO, the CEO’s humor resulted in immense stress (‘*it was so uncomfortable,’ ‘it stressed me out,” I think I lost some hair’)* for the secretary. In the Korean context, a joke (regardless of the joker’s intentions) by the senior manager can create distress for subordinates with an obvious negative affect on their psychological well-being.

However, an interview with Truscene’s CEO suggests that managers in high-ranking positions perceive the relationship between humor and emotions differently to their subordinates and they deem that it is the subordinate’s responsibility to be ‘not hurt’ in humor interactions:

You can hurt someone else’s feelings, if you don’t have enough understanding of that communicating person […] As an individual, you need to be able to look out for other people’s discomfort, but also you have to consider how uncomfortable the joking person will feel, if I react in an uncomfortable way because of my own background. So you should really say out loud “I feel uncomfortable or hurt when you joke like that. It would be appreciated if you could avoid jokes in that manner.” That will really complete your relationship. If you can develop your relationship like this, you can avoid being hurt. My recent thoughts are that people who feel hurt from humor are the problematic ones, rather than those who do the humor.

40–44, CEO

This example gives us mixed messages in how humor is perceived. Although the CEO recognizes that the use of some humor may hurt other people’s feelings, he also suggests that ‘*My recent thoughts are that people who feel hurt from humor are the problematic ones, rather than those who do the humor.’* The CEO believes that the victims of humor should try to let others know about their feelings and thus avoid being hurt from humor interactions. This illustrates the ambiguity and complexity created by the different perspectives and expectations between employees of different hierarchical positions here. Therefore, it is suggested that adequate responses toward humor needs to be made by individuals, depending on their relative roles (category 1a ‘fulfill relative roles,’ Table 3). The onus for responsibility in humor and for the psychological impact is not on the ‘joker’ as is usual in Western contexts, but seems to be influenced by the traditional Confucian hierarchical values and relationships in this Korean context. Thus, the responsibility falls upon the target of the joke, the subordinate, to manage their response in an ‘unproblematic’ way.

### Being Respectful and Preserving Harmony

Some of the participants felt that humor creates more distress for organizational members than relief in their Korean workplaces. In particular, many younger workers in lower hierarchical positions suggested that they experience more difficulties with contentious humor used by senior managers. This participant identifies that respect toward elderly people (filial piety) is important in Korean society and within the workplace (category 2b ‘being respectful,’ Table 3):

When it comes to communicating with a superior, you need to be careful. In Korea, you need to respect elderly people. Be polite. Things like that are considered important. And I agree… so I do things carefully. Including humor.

25–29, Mintrack

Although humor occurs, employees are highly restricted in their use of humor toward their organizational and societal ‘superiors’ or managers (‘*so I do things carefully. Including humor’)*. In this context ‘superiors’ includes individuals in higher organizational positions and also those older in age (‘*you need to respect elderly people’)*. The statement ‘*In Korea* […] *things like that are considered important’* suggests that respect toward elderly is strongly emphasized in Korea, and is considered common within the wider Korean society.

This participant discusses the expectations involved in responding adequately to a superior’s humor, even though Mintrack is the least formal and hierarchical of companies:

What can I say, he’s the boss? Of course I respond actively (I laugh) to the boss. But it’s not really that funny… well, if the same joke is shared with people my own age or position, and comparing the same joke shared with people positioned higher than me… it’s different.

25–29, Mintrack

Individuals in subordinate positions are expected to respond adequately (‘o*f course I respond actively (laugh) to the boss’)* even when the joke is not funny (‘*but it’s not really that funny’).* This means that an individual’s response to a humor interaction is heavily influenced by the joker’s status, and thus employees must consider the relative hierarchy between the communicators.

This consciousness toward hierarchy and effort toward maintaining respectful relationships means that humor tends to be single directional- usually the manager creates a joke and the subordinates must respond in a respectful, approving manner. Even if the manager’s joke is not comfortable, the subordinates cannot express their discontent as discussed by this employee:

There’s an old saying, you can’t spit on a smiling face. So you make them smile by smiling and laughing first. That usually gives them a favorable impression as well.

30–34, Mintrack

The proverb ‘*you can’t spit on a smiling face’* means that if you treat someone nicely, you would also avoid being ill-treated. Employees perceive that managerial humor is an appreciative behavior that creates a peaceful atmosphere and offers relief at work. Humor is seen here as ‘favorable,’ non-aggressive and a way of achieving the important Confucian state of ‘harmony’ amongst the collective. This desire to avoid direct conflict reinforces the traditional Korean value of collective harmony (category 2a ‘maintain sound relationships,’ Table 3).

### Negative Emotions

Many of the interview participants (29) discussed how workplace humor can provoke negative emotions. Most of these participants were positioned at lower hierarchical levels within the organization. Negative emotions can include being stressed by the confusing message conveyed in humor, discomfort in seeing another person being victimized through humor (category 3b ‘stress and discomfort,’ Table 3), or feeling hurt through (a direct) targeted humor (category 3a ‘feeling hurt and rejected,’ Table 3). These negative feelings create stress and tension for our participants. Here sexual jokes by one of the managers (targeted at female members or females in general) create discomfort for this male interviewee:

You would know who I am talking about. He’s always joking around, so people don’t think seriously of him. But when he’s with male workers, his jokes get a bit too far. The sort of ‘humor’ he makes toward women, and sometimes these are female workers from our company that he targets. Thank goodness that he doesn’t say it in front of her and only in front of us (all-male work team). I feel tainted, it’s really uncomfortable, and I want to wash my ears out after hearing his ‘jokes’.

30–34, Truscene

The respondent suggests that this humor creates discomfort and stress, even if he is not the targeted, and he feels ‘tainted.’ Stress is created in this situation where the employee perceives that he is socially (and culturally) expected to listen and respond to the joker’s (team leader’s) humor regardless of his emotions, and the hierarchical imbalance means that he cannot express his discomfort (category 3b ‘stress and discomfort,’ Table 3).

Here, being directly targeted in humor hurts this employee’s feelings, and she expresses her confusion and tension:

Something that I recall about humor at work is… well… rather than saying that it’s been bothering me, I sometimes wonder whether I have changed. Have I really become what everyone is saying? My colleague (ex-employee) said to me before he exited the company, is that I’m ‘stone-headed’. So along the same line [laugh] people keep on saying that I’m a stupid, but a nice person. So these days, I keep on thinking whether I’m really stupid, and sometimes people also say that I act fake. You heard it a couple of times, right? But it’s like, I wonder whether I’m just acting fake, or is it just my personality? I was thinking like that before. My younger sister behaves the same and I’m like this so… but if I think about it, I might actually look like a real flirt to them. But well, I keep on thinking to myself that people are just making fun of me, but again thinking that they are just joking, just joking, just joking, thinking like that over and over actually made me feel worried that, what if they actually think of this for real, well that kind of way […] it doesn’t really feel good, but I do laugh it off…it doesn’t feel good.

25–29, Mintrack

This example comprehensively illustrates the category ‘feeling hurt and rejected’ (3a, Table 3), and captures the mixed feelings that employees can experience through humor. This female employee is the youngest organizational member at Mintrack and has become the victim (or target) of on-going workplace humor. As she is the lowest in the organizational hierarchy, others have felt free to label her stupid and fake but only in ‘jokes.’ She does not accept this as ‘*just humor*’ but questions the underlying meaning. The humor is repetitive and ambiguous as she wonders: ‘*these days, I keep on thinking whether I’m really stupid.’* She also tries to persuade herself to see this as ‘just humor’ as we can see in her worried reflection: ‘*thinking that they are just joking, just joking, just joking, thinking like that over and over actually made me feel worried.’* The workplace humor that has denigrated and labeled her has resulted in significant ‘*worry*’ and created only negative emotions that she realizes, ‘*doesn’t feel good.*’ Additionally, she has felt compelled to ‘*laugh it off*’ creating further psychological distress and emotional dissonance as she hides her true feelings. Observations extend this reflection where our respondent is again the subject of humor teased by her managers at a café during lunch break:

Ivory, Diamond, Emerald and our researcher sit around the square table, each drinking their iced coffee. Ivory is the subordinate employee in this group. Emerald asks questions about the researcher’s home and country until Ivory suddenly widens her eyes and blurts out:

Ivory: Bananas! I saw once on TV, aren’t bananas overseas green? And not yellow? And you cook them rather than eating them raw?

Emerald looks at Ivory shakes her head sideways and covers her face with one hand.

Emerald: Oh my god, I am so embarrassed, I think you just watched the TV program ‘The Law of the Jungle,’ not any educational TV programs.

Diamond laughs, then quickly frowns again.

Diamond: I think you are settling with the ‘nice but stupid person’ image. You are seriously stupid.

Emerald: Are you doing that on purpose, Ivory?

Ivory laughs, but her face turns red. She starts to gaze around the café, as if not to make eye contact with anyone. Ivory then turns around to face me (researcher), and whispers in my ear:

Ivory: It feels like I’m on a minefield. Bombs everywhere.

April 18, Mintrack Observation notes

This observation exemplifies how Ivory is jokingly criticized by managers. She is openly, but jokingly called ‘stupid’ due to her incorrect knowledge about banana farms. Ivory’s behavior (face turning red, gazing round the cafe) suggests that she is embarrassed. Her comment about the bombs and suggests that she feels that the humor used to define her as stupid, is threatening and harmful but she accepts the humor without complaint.

This employee’s words suggest that humor creates anxiety through having unpredictable consequences:

It makes me feel anxious. Sometimes people overdo jokes, and it’s mischievous, and confusing. The consequences are quite unpredictable, but they do it anyway.

40–44, Mintrack

This example also identifies negative emotions created through humor. Our participants described humor as: ‘confusing,’ ‘unpredictable’ and ‘mischievous,’ which can make employees feel anxious. Regardless of these negative emotions, humor still operates consistently in these workplaces (‘*but they do it anyway’*) and it seems that because humor is interpreted in various ways, our participants accept humor interactions but make their own interpretations of the value of humor at work. Their perceptions may be based on the reaction that they feel compelled to display in order to satisfy hierarchically ‘superior’ managers and colleagues. While humor may create confusion for some individuals, it may also emotionally damage others. In particular, those in lower hierarchical positions seem more often subject to targeted humor, but are unable to express their discomfort due to the hierarchical relationship structure and the cultural expectation to remain respectful toward their superiors. The conflict between cultural expectations and individual’s perceptions may lead to further stress and negative emotions as well as happiness, warmth and favorable impressions. These complex tensions underpin our discussion as we attempt to answer our research question next.

## Discussion: Emotionally (UN) Well?

Many past studies (e.g., Samson and Gross, 2012) have emphasized the positive emotional effect of humor. Positive humor is important in organizational studies, as feelings of enjoyment and happiness created in humor can enhance communication (Alden et al., 1993; Holmes, 2000) and develop workplace relationships (Martin, 2004). Employees may also use humor to indirectly express their minds and release stress, in order to manage their own emotions (Freud, 1960). Therefore, humor has been considered a method or device used to improve the psychological well-being, mental and physical health of workers, (Erickson and Feldstein, 2007; Crawford and Caltabiano, 2011; Papousek, 2018). Humor styles have been positioned as ‘light’ and therefore can enhance the self and be affiliative or ‘dark’ which can include aggression and be detrimental (Martin, 2003). However, few prior studies offer examples where both styles may operate simultaneously and offer some fun and pleasure for some people but cause distress for others.

Our participants in the current study suggested that humor may help to release stress and tension. However, many of the research participants in lower hierarchical positions also suggested that humor may also lead to negative emotional effects such as hurt feelings and anxiety. It is important to note that instances of humor can have both light and dark aspects at the same time. For example, a superior may make a joke that many of the subordinates enjoy and a collective good feeling may be created. However, one subordinate may find the humor offensive and/or insulting and therefore may experience psychological distress. Adding to this distress is the need to either smile or remain quiet, but overall to accept the humor in the interests of collective harmony. This ambiguity and tension is exemplified by one of the youngest members in Mintrack who expressed her ‘*worry’* about her superiors’ use of workplace humor. Due to her low (organizational and hierarchical) status, she stated that she was unable to express her truthful feelings and reactions to her superiors’ humor. Instead, this employee showed a complicated mix of emotions, of feeling hurt through being the target of humor and her inability to respond honestly but she was also relieved that to be included in humor interactions. In the interests of collective well-being she did not show her hurt and confusion but displayed the expected normative behavior and she quietly tolerated the humor.

Smith and Powell (1988) study suggests that managers’ disparaging (dark) humor is rather disliked by subordinates, and is perceived as an unsuitable method to relieve tension. This means that superiors’ use of disparaging humor may damage relationships with subordinates by creating adverse emotions. Furthermore, such use of humor may be considered aggressive (and not ‘*just humor’*) regardless of the intentions behind the humor. Extending Smith and Powell’s (1988) findings, where the hierarchical (positional) difference between organizational members may influence the effects of humor, the damaging impact of humor may be more dramatic in cultural contexts (such as Confucian contexts) which have greater underlying hierarchical differences between individuals. Furthermore, the greater social power that superiors have over subordinates in Confucian contexts and in our findings implies that this emotional impact of humor may be greater on those in subordinate positions, to reflect this workplace dynamic.

Similarly, Gilbreath and Benson (2004) suggest that an employee’s psychological well-being is strongly influenced by the behavior of their supervisors, and Richman et al.’s (1996) study suggests that managers’ aggressive humor may lead to behavioral and mental health problems among workers. Supporting this view, Ruch et al. (2018b) also suggests that mockery humor styles relates to lower psychological well-being of individuals, and this implies that such styles of humor may emotionally impact interacting individuals more negatively than intended. While this phenomenon is not necessarily limited to Confucian contexts, those with less power within any relationships that embeds an imbalance of power between individuals (such as manager–employee relationships), may be constrained from expressing any discontent toward inappropriate interactions, due to their dependency on the superior/manager (Langan-Fox et al., 2007; Tepper et al., 2009). Therefore, manager-subordinate humor interactions experienced as aggressive by subordinates may create only distress for them rather than the enjoyment expected by managers.

Our observations suggest that hierarchical differences may be related to the emotional damage or stress subordinates experience in some humor interactions. This means that the potential stress an employee feels from sharing humor with the CEO may be even greater to that felt when interacting with a manager. Many Korean companies are based on a seniority system, where individuals’ progress through the organizational hierarchy is based on age and tenure (within the company). This linkage between age and organizational hierarchy also suggests that the level of stress gained in a humor interaction may be influenced by age differences. For example, a young employee at Mintrack explained that it is stressful to respond to the CEO’s humor, as he has both positional status (‘*he is the CEO’*) and is much older than her- both aspects influencing the response she feels compelled to make. All of our subordinate participants suggested that they experienced humor interactions with those of greater age/status to be more stressful. This may be because the cultural values and expectations are changing within Korean society, and younger Koreans are moving away from the more traditional Korean values (Ingelhart, 1997). This suggests that humor boundaries may be changing and older managers who feel unconstrained in their humor are at risk of continually upsetting subordinate workers with changing values influenced by Western experiences, as they adopt less- traditional perspectives and behaviors. Emotional responses to humor and the dissonance experienced by our subordinate, younger Korean workers may be predictive of future cultural clashes and on-going workplace disquiet.

Even though change may be approaching, Ingelhart and Baker (2000) argue that cultural heritage and traditions such as Confucianism strongly impact the values of the people regardless of the level of economic development within a society. In particular, the Confucian value of harmony is still strongly recognized and appreciated by the younger Korean people (Zhang et al., 2005). As seen in our findings, while the younger organizational employees in these Korean workplaces may be developing different views toward organizational humor, they still seem to value the importance of maintaining harmony within their workplaces which still restricts them from discussing their perspective when involved in humor interactions. As the Confucian value of harmony is based upon the maintenance of relational (hierarchical) roles between individuals, our data suggests that humor is most often created by superiors, and that individuals with higher status is perceived to have more legitimacy to initiate humor. Thus, subordinates may consciously avoid initiating humor toward their superiors, and also stating their true feelings (about the humor interaction) as this could create conflict with their managers. Therefore, lower level employees occupy an ambiguous position where the humor behaviors and different cultural values of their managers create distress for them. We argue that humor interactions may be a catalyst opening a divide between employees and managers and this changing dynamic highlight shifting cultural values in Korean workplaces and the subsequent impacts upon the psychological well-being of workers.

## Conclusion and Implications

This paper provides cultural interpretation to organizational humor and its influence on psychological well-being, within Korean organizations. Both dark and light humor may help to relieve stress and tension in Korean organizational contexts, but more we found that often humor interactions created distress and negative emotions for those in lower hierarchical positions. The Confucian value of hierarchy and harmony helps to shape organizational relationships and influence response to humor interactions within these Korean workplaces. Unequal relationships and employees’ relational and organizational status influence how humor interactions may be responded to- even when they are perceived and interpreted negatively. While humor may help to relieve stress and tension for Korean organizational members the hierarchical relationship structure, change in cultural values amongst different generations, and the value that harmony that younger Korean workers maintain, all potentially play an important part in shaping workplace humor interactions. Subordinate employees may experience on-going stress and tension from mangers’ humor, especially as they are unable to express their true feelings about some of the humor aimed at them. Such conflict and dissonance may affect employees’ psychological well-being and offers us unique cultural insights into the phenomenon of workplace humor. Our overarching contribution is in illuminating the humor dynamics and their implications for employee psychological well-being within this studied Confucian (Korean) organizational context. Cultural considerations of humor are not easily found in the extant literature which maintains a firm focus on mostly Western, conceptions and considerations of workplace humor.

There are various limitations to this research. First the nature of this qualitative research means that the data reflects the views of the particular studied companies and participants only, limiting the generalizability of the findings, and thus may not relate to all Korean organizations. Second, only three companies were investigated in this study. Researching a larger number of companies may help to provide more reliable and in-depth data on the studied phenomenon. Third, spending more time (longer than the 1-month period conducted in this current research) within the participant companies may help to gain furthe insights on humor interactions within Korean workplaces. Fourth, the presence of the researcher could have influenced some of the results, as the researcher participated and was involved in the organizational activities of all three researched companies. Last, the interpretative approach adopted in this study means that the researcher’s own beliefs and interpretations are embedded in the research process. However, we acknowledge this subjectivity involved within the research process, and that the researcher is considered as a part of the studied context (Alvesson, 2010) in the methodology adopted. While these limitations are acknowledged we were still able to explore the relationship between humor, culture, and psychological well-being in these specific Korean organizations. Our acknowledged limitations will guide and influence our research design for future projects that may build upon this initial exploratory cultural humor study.

## Ethics Statement

This study was carried out in accordance with the recommendations of University of Auckland Ethics Guidelines, The University of Auckland Human Participants Ethics Committee. The protocol was approved by The University of Auckland Human Participants Ethics Committee. All subjects gave written informed consent in accordance with the Declaration of Helsinki.

## Author Contributions

HK had overall responsibility for the design and conduct of the research, writing the literature review and analyzing the qualitative data. BP also conducted the analysis of qualitative data. HK and BP contributed to the writing of the manuscript.

## Conflict of Interest Statement

The authors declare that the research was conducted in the absence of any commercial or financial relationships that could be construed as a potential conflict of interest. The reviewer SL and handling Editor declared their shared affiliation at the time of review.
